# Acute heart failure due to autoimmune myocarditis under pembrolizumab treatment for metastatic melanoma

**DOI:** 10.1186/s40425-015-0057-1

**Published:** 2015-04-21

**Authors:** Heinz Läubli, Cathrin Balmelli, Matthias Bossard, Otmar Pfister, Kathrin Glatz, Alfred Zippelius

**Affiliations:** Division of Medical Oncology, University Hospital Basel, Basel, Switzerland; Division of Cardiology, University Hospital Basel, Basel, Switzerland; Institute of Pathology, University Hospital Basel, Basel, Switzerland; Department of Biomedicine, Cancer Immunology Laboratory, University of Basel, Basel, Switzerland

**Keywords:** Melanoma, Immunotherapy, PD-1 blockade, Antibody, Nivolumab, Pembrolizumab, Autoimmunity, Anti-tumor T cell response, Myocarditis

## Abstract

Antibodies that stimulate the immune system by targeting inhibitory T cell receptors were successfully introduced into oncological practice and are capable to overcome tumor-induced immune evasion. In particular, targeting of the inhibitory receptors CTLA-4 and PD-1 or its ligand PD-L1 have been shown to be beneficial for patients with melanoma, renal cell cancer, non-small cell lung cancer and a growing list of other cancers with impressive response rates. Here, we report a severe, potentially life-threatening side effect of anti-PD-1 immunotherapy with pembrolizumab, which has not been previously described in the literature. A 73-year-old woman with metastatic uveal melanoma treated with pembrolizumab in third line developed severe heart failure due to pembrolizumab-mediated autoimmune myocarditis. Echocardiographic studies revealed a severely impaired left ventricular function with dyssynchrony. All tests for cardiotropic viruses were negative and histological analysis of a myocardial biopsy showed lymphocytic infiltration with a predominance of CD8 positive cells and a reduction of FOXP3 positive regulatory T cells. After initiation of corticosteroids and guideline-conform heart failure therapy, the symptoms rapidly improved and the left ventricular function recovered. While autoimmune myocarditis is a documented side effect of other checkpoint inhibitors, as for example ipilimumab and in one case with anti-PD-L1 antibody, it is not described for anti-PD-1-antibodies like pembrolizumab or nivolumab. As the FDA recently approved both pembrolizumab and nivolumab for melanoma progressing after anti-CTLA-4 treatment with ipilimumab, more patients will soon receive anti-PD-1 therapy. Thus, it is important to be aware of such rare, but severe immune-related adverse events.

**Electronic supplementary material:**

The online version of this article (doi:10.1186/s40425-015-0057-1) contains supplementary material, which is available to authorized users.

## Background

The field of cancer immunotherapy is currently moving forward at an accelerated pace. Though early clinical trials have yielded mixed results with ambiguous clinical benefit [1], cancer immunotherapy is now attracting increasing attention as viable therapeutic option, both in melanoma and in other malignancies [2]. In particular, recent therapeutic efforts targeting inhibitory receptors on T cells to overcome tumor-induced immune dysfunction has been successfully introduced into oncological practice. The clinical development of immune checkpoint blocking antibodies has been pioneered by the antibody ipilimumab (Yervoy®), which inhibits CTLA-4 and has demonstrated survival benefit in two randomized landmark trials in melanoma [3,4]. Capitalizing on this success, research on clinically relevant T cell checkpoint inhibition has been boosted. Early clinical trials have demonstrated meaningful response rates, sustained clinical benefits with exceptional survival rates and good tolerability of next-generation checkpoint inhibitors, including PD-1 and PD-L1 inhibitors across multiple cancer types [5-10]. Exciting perspectives include the concurrent blockade of different immunologic (non-redundant) checkpoints. The feasibility of this approach has recently been demonstrated in melanoma using combined CTLA-4 and PD-1 inhibition [11].

Inhibition of immune checkpoints induces side effects defined as “immune-related adverse events” (irAEs) [12]. Autoimmunity is the suggested mechanism sustaining these toxicities. Such irAEs often affect the skin, the intestinal mucosa or endocrine organs. Here, we report a case of autoimmune myocarditis with consecutive heart failure after treatment targeting PD-1.

## Case presentation

**Figure 1:**
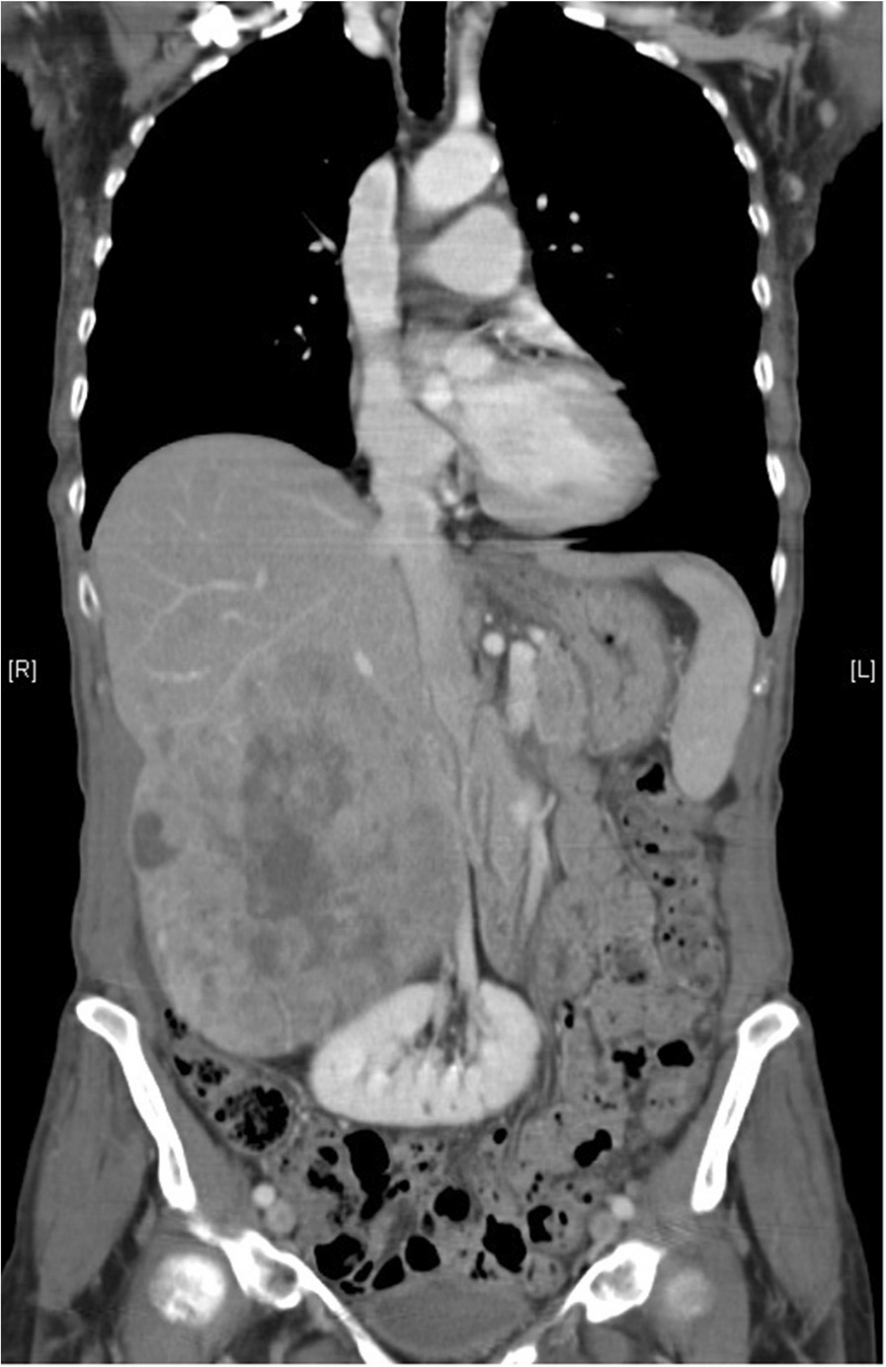
**Computed tomography at initiation of pembrolizumab.** Computed tomography scan of the liver before treatment with pembrolizumab was initiated after progression with the treatment of ipilimumab.

A 73-year-old woman with metastatic uveal melanoma presented for a clinical examination and laboratory control under third-line therapy with pembrolizumab (MK-3475) in our oncological outpatient department. She suffered from progressive, severe dyspnea. Fourteen years earlier, a malignant uveal melanoma of her left eye was diagnosed and treated by ablative proton therapy. In February 2013, a relapse with hepatic metastases was diagnosed. No BRAF or NRAS mutations were found in the liver biopsy in accordance with low frequency of such mutations in uveal melanoma. The patient had no prior history of autoimmune or allergic disease. The hepatic lesions showed progressive disease after treatment with two cycles of dacarbazine, and, subsequently, four cycles with the anti-CTLA-4 antibody ipilimumab. Importantly, the latter therapy was tolerated without any adverse events. To improve local control, selective internal radiation therapy (SIRT) of the largest liver lesions was performed in December 2013. However, computed tomography 6 months later showed new bone, pulmonary, mesenterial and peritoneal metastasis (Figure 1). Palliative radiation therapy of symptomatic, lytic bone lesions was performed and denosumab monthly was started. Moreover, anti-PD1 therapy with pembrolizumab was initiated one month later at a dose of 2 mg per kilogram body weight every third week, which led to stabilization of the disease after 8 weeks. The patient had no history of cardiac disease.

**Figure 2:**
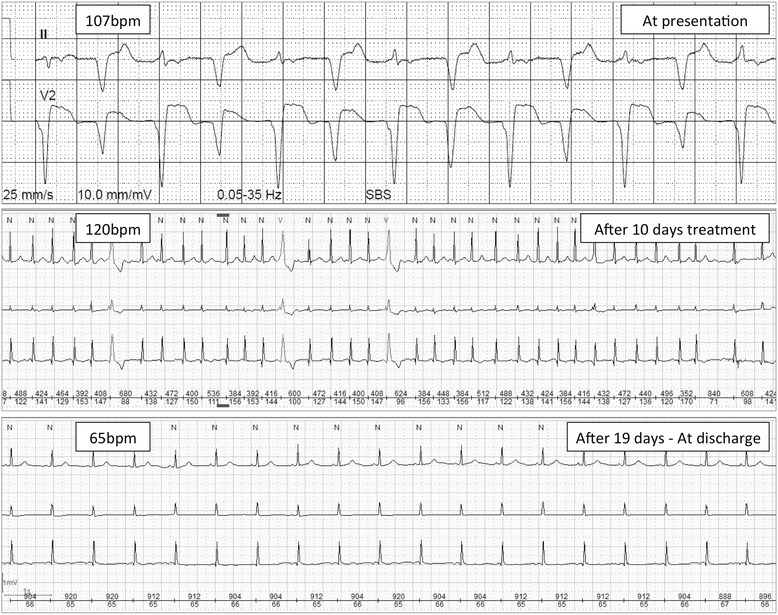
**Electrocardiograms – At presentation and during follow-up.** The electrocardiograms at admission, after 10 and 19 days of treatment are presented.

When she presented with progressive dyspnea (New York Heart Association Stage (NYHA) IV), she had received five cycles of pembrolizumab. Her prior medical history was free of any heart diseases or corresponding symptoms. Clinical examination now revealed congested neck veins, bilateral rales and lower leg edema. The electrocardiogram (ECG) showed a tachycardiac sinus rhythm with ventricular bigemy (Figure 2). Brain natriuretic peptide (BNP) and high-sensitivity troponin T (hs-TnT) levels were elevated (928 ng/L and 0.63 μg/L, respectively). Thyroid stimulating hormone levels were within normal range (2.65 mlU/l). A pulmonary embolism or pneumonitis was excluded by computed tomography. The transthoracic echocardiography revealed a severely impaired left ventricular ejection fraction (LVEF) of 30% with marked ventricular dyssynchrony (*Movie Clip*, Additional files 1-3). Taken together, the clinical presentation corresponded to acute heart failure.

**Figure 3:**
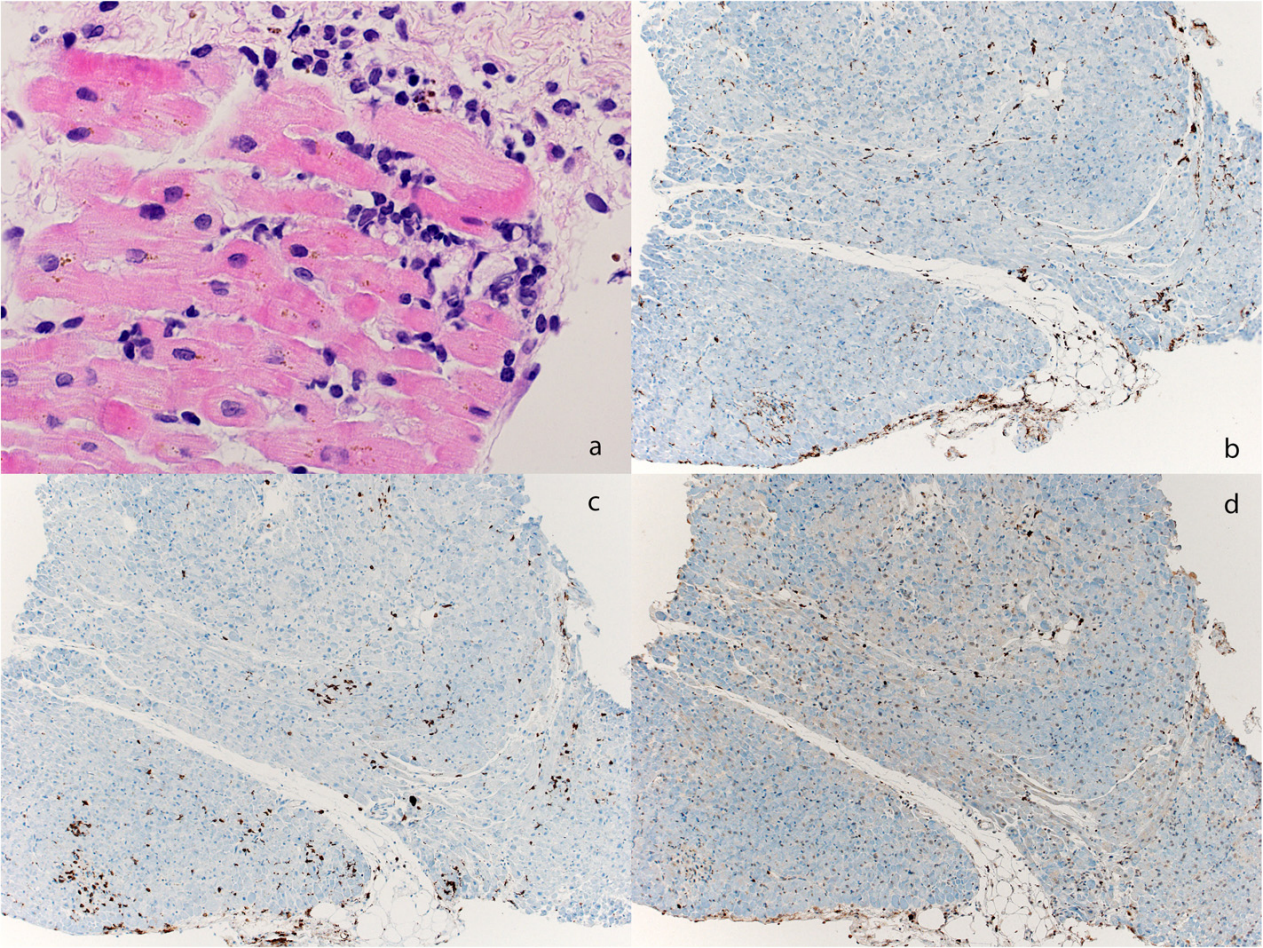
**Histological analysis of endomyocardial biopsy. (a)** Hematoxylin and eosin staining of the myocardial biopsy with focal mononuclear infiltrates. **(b)** Immunohistochemical analysis of CD68 macrophages. **(c)** Staining for CD8 positive T cells **(d)** and FOXP3 positive cells within the myocardium of the patient.

The cardiac MRI did not show any signs consistent with an acute myocarditis or myocardial ischemia. Confronted with an unclear acute heart failure under immunotherapy, we performed a myocardial biopsy, which eventually confirmed the diagnosis of a lymphocytic myocarditis. An infiltration predominantly CD8 positive T cells was noted (Figure 3). Only a few FOXP3 positive, regulatory T cells were found showing signs of apoptosis (Figure 3). Serological and in situ analyses for cardiotropic viruses were negative. Thus, the diagnosis of immune-mediated myocarditis as irAE under pembrolizumab treatment was rendered.

We started a therapy with an AT2-receptor blocker (candesartan), a beta-blocker (bisoprolol), aldosterone-antagonist (spironolactone) and diuretics (torasemid) according to ACC/AHA guidelines as well as prednisone 2 mg per kilogram body weight. Within two weeks, our patient showed a significant symptomatic recovery (NYHA II). This was in accordance with a normalization of the ECG (Figure 2) and echocardiographic improvement of left ventricular function (mildly reduced LVEF, 52%). Also BNP and hs-TnT levels had significantly decreased (154 ng/L and 0.075 μg/L, respectively). The patient could be discharged and anti-PD1 therapy was halted. No other specific anti-tumor therapy was initiated at the time.

## Discussion

On September 4, 2014, the FDA granted accelerated approval of pembrolizumab (KEYTRUDA®) for the treatment of patients with unresectable or metastatic melanoma and disease progression following ipilimumab after the analysis of an expansion cohort of the KEYNOTE-001 trial of 173 patients [6]. The 2 mg and 10 mg per kilogram body weight regimens every 3 weeks showed similar response rates. Drug-related adverse events of any grade occurred in 82% and only 12% had grade 3 or 4 adverse events [6]. Grade 3 or 4 side effects due to immune stimulation or irAEs were only noted in 3 patients and included autoimmune hepatitis, maculopapular rash and pancreatitis [6]. In earlier studies of pembrolizumab, 4% of patients had pneumonitis, 1% grade 3 aminotransferase elevation, 8% hypothyroidism, 2% potentially autoimmune-mediated renal failure and one patient had autoimmune adrenalitis and hyperthyroidism [8]. Diarrhea developed in 20% of patients, but was usually mild and could be controlled without glucocorticoids [8]. Toxicities were less frequent in patients receiving 2 mg per kilogram body weight every 3 weeks compared to 10 mg every two or three weeks [8]. The toxicity profile and frequency of irAEs under pembrolizumab alone compared favorable to anti-CTLA-4 treatment with ipilimumab alone or in combination with anti-PD-1 therapy with nivolumab [11,12]. Similarly, pneumonitis, a potentially life-threatening complication, was noted less frequently with pembrolizumab than in studies using nivolumab alone [10], although the tumor type likely plays a role in the toxicity profile. Future studies in other tumor types and combinations of immunostimulatory therapies that include pembrolizumab will certainly require careful monitoring and dose escalation schemes to avoid severe irAEs. Temporary or definitive discontinuation of immunostimulatory treatment and temporary immunosuppression can be an effective treatment in most cases and safety management guidelines are implemented in clinical trials and should also be used for patients treated outside trials [7].

There is no prior report on perimyocarditis or endocarditis after treatment with pembrolizumab [6,8]. While a similar spectrum of toxicities was observed with anti-PD-1 antibody nivolumab [5,7] and anti-PD-L1 antibody [9,13-15], only one case of myocarditis was reported in a phase one trial testing anti-PD-L1 antibody [9]. Myocarditis of non-infectious cause was previously described with anti-CTLA-4 treatment [3]. Histological analysis of autoimmune lesions and tumors after treatment with immune checkpoint inhibitors usually shows infiltration of effector CD8 T cells and reduction of regulatory FOXP3 positive T cells similarly as seen in the analysis of the myocardial biopsy in our patient [16] (Figure 3). Interestingly, various tissue-specific autoimmune conditions are observed in mice deficient for PD1 (*Pdcd1*^*−*/*−*^) [17]. These mice, particularly with a Balb/c background, die of heart disease, which is similar to human dilated cardiomyopathy. There is almost no inflammation in the heart of these mice and subsequent analyses revealed that auto-antibodies against cardiac troponin I are responsible for the disease [18]. In mice that are genetically predisposed to systemic autoimmunity, PD1 deficiency results in fatal myocarditis by 10 weeks of age that is reminiscent of *Ctla4*^*−*/*−*^ mice [19]. Massive infiltration of both CD4 positive and CD8 positive T cells and myeloid cells was found in hearts of those mice concomitant with the production of high-titer auto-antibodies against cardiac myosin. Subsequent experimental work clearly confirmed the important role for PD-1 in protecting the heart from T cell-mediated damage [20]. PD-1–deficient T lymphocytes caused enhanced disease with increased cytotoxic activity and inflammatory infiltrate.

Differential diagnosis of lymphocytic myocarditis and dilated cardiomyopathy includes infections with cardiotropic viruses. Among viral causes, enteroviruses and adenoviruses are historically common causes, but more recently, parvovirus B19, the recent H1N1 influenza pandemic, and human herpes virus 6 have become more prominent [21]. Although it carries some inevitable limitations, the endomyocardial biopsy remains the gold standard for the diagnosis of myocarditis [22]. According to the Dallas criteria, myocarditis is defined by lymphocytic infiltrates with or without myocyte necrosis. Lately, these criteria were challenged due to their limitations, including low sensitivity and high interobserver variability in interpretation of biopsy samples [22,23]. Therefore, advances in immunohistochemistry and PCR analyses of the endomyocardial specimens improved diagnostic accuracy for myocarditis [22,23]. In the presented case, we performed extensive diagnostics and excluded a viral cause for myocarditis. It is therefore very likely that the myocarditis of the patient was a result of immune stimulation due to PD-1 blockade with pembrolizumab.

## Conclusion

We report here an autoimmune myocarditis as a side effect of an anti-PD-1-antibody, completely resolving after a therapy with high-dose corticosteroids. To our knowledge, it is the first time an autoimmune myocarditis under pembrolizumab treatment is reported. It is a documented side effect of other checkpoint-inhibitors, as for example ipilimumab and in one case with anti-PD-L1 antibody, but not in anti-PD-1-antibodies like pembrolizumab or nivolumab. Our report should raise awareness for *de novo* cardial dysfunction in patients under PD-1 blockade. Approval of pembrolizumab and nivolumab by the FDA for the treatment of melanoma will lead to the use of these antibodies in a broader patient population with more concomitant diseases. Further ongoing studies and experience with patients outside of trials will provide more information about such rare side effects.

## Consent

Written informed consent was obtained from the patient for publication of this case report and any accompanying images. A copy of the written consent is available for review by the Editor-in-Chief of this journal.

## Additional files

Additional file 1:
**Transthoracal echocardiography at the time point of diagnosis of acute heart failure due myocarditis.** Movie clip of apical 4 chamber (A4C) view. Additional file 2:
**Transthoracal echocardiography at the time point of diagnosis of acute heart failure due myocarditis.** Movie clip of parasternal long axis (PLAX) view. Additional file 3:
**Transthoracal echocardiography at the time point of diagnosis of acute heart failure due myocarditis.** Movie clip of parasternal short axis (PSAX) view.
